# Reduced Fluoroscopy Time With Physician-Controlled Fluoroscopy During Endoscopic Retrograde Cholangiopancreatography: A Community Hospital Experience

**DOI:** 10.7759/cureus.13771

**Published:** 2021-03-08

**Authors:** Samir Kakodkar, Ali Haider, Ryan T Hoff, Ina Zamfirova, Kenneth Chi

**Affiliations:** 1 Department of Medicine, Division of Gastroenterology, Advocate Lutheran General Hospital, Park Ridge, USA; 2 Russell Research Institute, Advocate Lutheran General Hospital, Park Ridge, USA

**Keywords:** ercp, endoscopic retrograde cholangiopancreatography, radiation exposure, fluoroscopy time, physician, fluoroscopy intervention, fluoroscopy, ercp complications

## Abstract

Background and objective

Fluoroscopy during endoscopic retrograde cholangiopancreatography (ERCP) is associated with radiation exposure and related health risks. Either the physician or the radiology technologist can activate fluoroscopy during ERCP. The aim of this study was to determine if physician-controlled fluoroscopy is associated with decreased fluoroscopy time, which may correspond to less radiation exposure to patients and staff.

Methods

We conducted a single-center, retrospective study; data were collected on ERCP performed using physician-controlled and technologist-controlled fluoroscopy. Fluoroscopy time, procedure complexity level, and Stanford Fluoroscopy Score were compared between the two groups.

Results

The median fluoroscopy time significantly differed between the two groups with 108 seconds for physician-controlled and 146 seconds for technologist-controlled procedures (p=0.004). The ratio of median fluoroscopy time to procedure complexity level was significantly lower in the physician-controlled group at 73.0 seconds compared to 97.0 seconds in the technologist-controlled group (p=0.002). The ratio of median fluoroscopy time to Stanford Fluoroscopy Score was 25.5 seconds in the physician-controlled group compared to 39.3 seconds in the technologist-controlled group, which was also statistically significant (p<0.001). A subgroup analysis of physicians with advanced training in ERCP also showed a significantly reduced median fluoroscopy time to Stanford Fluoroscopy Complexity Score ratio: 25.5 seconds for physician-controlled versus 35.0 seconds for technologist-controlled (p=0.001).

Conclusion

The ERCP technique with physician-controlled fluoroscopy may be associated with shorter fluoroscopy time. This may correspond to decreased radiation exposure to patients compared to radiology technologist-controlled fluoroscopy. Further investigations with larger, prospective studies are warranted.

## Introduction

Endoscopic retrograde cholangiopancreatography (ERCP) is a diagnostic and therapeutic procedure utilized in patients with pancreatic and biliary tract diseases. The physician performing the procedure (endoscopist) uses a fluoroscopic system in order to obtain real-time images that aid in the diagnostic and therapeutic interventions. The use of fluoroscopy introduces the risks of radiation exposure to the patient and also to the physician, radiology technologist, nurses, and staff present in the procedure room. The risks of radiation exposure include the development of cataracts, dermatitis, and cancer [1]. From 1980 to 2006, the per capita medical radiation exposure in the United States increased six-fold [2]. However, in recent years, radiation exposure has decreased, which may be due in part to awareness of the radiation dose and efforts to minimize radiation exposure [2].

Several settings are available to decrease the amount of radiation exposure in an ERCP procedure. Low dose rate fluoroscopy should be used whenever possible and a pulsed fluoroscopy mode with the lowest frame rate setting that achieves adequate image quality is advisable [3]. Protective lead aprons and thyroid shields should be worn by everyone in the procedure room when X-rays are used, and protective lead glasses should be used by staff nearest to the patient [4,5]. The patient should be positioned as far away from the X-ray tube as possible, and collimator blades manually adjusted to only include the area of interest [6]. A recent multivariate analysis of ERCP suite ergonomics showed that decreasing the distance between endoscopy and fluoroscopy screens significantly reduces fluoroscopy time [7].

In 2006, the American Society of Gastrointestinal Endoscopy (ASGE) and the American College of Gastroenterology (ACG) proposed quality indicators for ERCP, which focused on appropriate indications, technical success, and adverse events. Notably, radiation exposure or an appropriate proxy to measure it was not included as quality indicators. More recently, including fluoroscopy time as one of these quality measures has been recommended [8,9]. In 2015, the ASGE and ACG quality indicators were updated, with a recommendation to measure and document fluoroscopy time [10]. In 2009, the World Gastroenterology Organisation Global Guideline recommended limiting radiation exposure during all procedures to levels "as low as reasonably achievable" (ALARA) [11]. According to the International Commission on Radiological Protection, when fluoroscopy is necessary, the aim should be to complete the procedure in the shortest duration possible [3].

Monitoring fluoroscopy time has been associated with a reduction in radiation exposure, and any factors that increase the time of radiation exposure, including fluoroscopy time, will increase the radiation dose and risks associated with ionizing radiation [3,12]. Multiple factors have been shown to affect fluoroscopy time, including procedure difficulty, trainee involvement, physician experience, physician endoscopy volume, and particular therapies [12-16]. A prospective randomized trial showed that time-limited fluoroscopy where X-ray exposure is limited to three seconds every time the pedal is pushed is associated with 16.4% lower fluoroscopy time [17]. As radiation exposure to patients increases in the US and worldwide, it is pertinent to study any variables that may affect radiation exposure, particularly ones that can be modified directly by the physician.

There exists a linear relationship between fluoroscopy duration and radiation dose, and a reduction in radiation exposure occurs with the monitoring of fluoroscopy time [5,18,19]. Fluoroscopy time is the most important factor affecting the radiation dose to the patient [15]. Fluoroscopy is typically controlled by either the physician via foot pedal or a radiology technologist via hand remote. Both methods of fluoroscopy control are used in clinical practice; however, the exact prevalence of each is unknown. To our knowledge, no published studies have directly compared both methods. The aim of this study was to determine if physician-controlled fluoroscopy is associated with decreased fluoroscopy time. The primary endpoint of this study was the amount of fluoroscopy time in physician-controlled and technologist-controlled fluoroscopy during ERCP procedures.

Preliminary results from this study were presented at the 2016 American College of Gastroenterology Annual Scientific Meeting in Las Vegas, Nevada, October 14-19, 2016 [Presentation: Kakodkar S, Haider A, Hoff RT, Chi K. Reduced Radiation Exposure with Endoscopist-controlled Fluoroscopy During Endoscopic Retrograde Cholangiopancreatography (ERCP). 2016 American College of Gastroenterology Annual Scientific Meeting; October 18, 2016].

## Materials and methods

We conducted a retrospective comparative study, which included adult patients undergoing ERCP procedures at an independent, community-based 638-bed teaching hospital between January 2011 and February 2015. Retrospective data were collected from a time period preceding the disclosure of the study to the participating physicians. Pregnant patients, prisoners, and children were excluded from the analysis. ERCPs with fellow participation were also excluded in order to reduce variability in fluoroscopy and procedure length from the analysis. The diagnostic or therapeutic success of the procedure did not affect inclusion in the study. Data were collected from two groups of patients that made up the study sample: those who underwent physician-controlled fluoroscopy (cases) and those who had radiology technologist-controlled fluoroscopy (controls). The cases included one physician with advanced training in ERCP who controlled fluoroscopy with a foot pedal, while eight physicians, two of whom had advanced training in ERCP, utilized a technologist to control fluoroscopy. The selection was based on physician preference. All participating physicians were fellowship-trained gastroenterologists. No physician utilized both technologist and physician-controlled fluoroscopy. All cases were performed with Pentax Medical Video Duodenoscopes and mobile C-Arm Fluoroscopy Machines (Model 9800, SN 82-1888, General Electric, Boston, MA). In all cases, a technologist was present at the bedside to assist in positioning the X-ray machine, if necessary. All radiology technologists were credentialed and registered by the American Registry of Radiologic Technologists (ARRT) and the Illinois Emergency Management Agency (IEMA) and had satisfied continuing education requirements to maintain credentialing.

Advanced training in ERCP was defined as any additional formal training in ERCP beyond a general two-to-three-year gastroenterology fellowship. Physicians were classified into three groups: low-volume (<50 ERCP cases/year), moderate-volume (50-200 ERCP cases/year), and high-volume endoscopists (more than 200 ERCP cases/year); this was determined by the average number of ERCPs performed at the study center over a five-year period. Clinical assessment tools, such as direct observation of procedural skills (DOPS), are not routinely used at this hospital following the completion of formal training.

In rare cases, magnification was used, although a majority of ERCPs were performed using the same machine with identical settings, which were set up by a radiology technologist for all patients in both groups. Lead drapes on the c-arm and in-ground fluoroscopy were not used. Radiation dosimeters were not used. Radio-opaque screens between the endoscopist and the fluoroscope were not used. In all cases, fluoroscopy and endoscopic monitors were positioned next to each other. Fluoroscopy time was not displayed for physicians or technologists in either group. Time-limited fluoroscopy was not used in any case.

The endoPRO iQ® (PENTAX Medical, Montvale, NJ) electronic medical record was used for extraction of endoscopy reports, which routinely include fluoroscopy time and procedure time. Fluoroscopy time was defined as the start of fluoroscopy until the end of fluoroscopy, which was cumulative. The sum of fluoroscopy time was recorded routinely in the procedure report. Procedure time was defined as the time from the insertion of the endoscope into the patient’s oropharynx, until the removal of the endoscope.

All procedures were graded on procedural complexity using the procedure complexity level, previously described by Heyd et al. [15]. Diagnostic and therapeutic procedures performed during ERCP were assigned a complexity level from 1 to 4, with the higher numbers indicating greater procedure complexity. If the procedure was completed outside of normal working hours or if the procedure had been attempted unsuccessfully before, then the level increased by one. Level 1 was assigned for deep cannulation of the bile duct, biliary stent exchange, or removal. Level 2 was assigned for biliary stone extraction of <10 mm, treatment of biliary leaks, and treatment of extrahepatic benign and malignant strictures. Level 3 was assigned for biliary stone extraction of >10 mm, minor papilla cannulation in divisum, removal of internally migrated biliary stents, intraductal imaging or biopsy or fine-needle aspiration (FNA), management of acute or recurrent pancreatitis, treatment of pancreatic strictures, removal of mobile pancreatic stones of <5 mm, treatment of hilar tumors, treatment of benign biliary strictures (hilum and above), and management of unsuspected sphincter of Oddi dysfunction. Level 4 was assigned for removal of internally migrated pancreatic stents, intraductal image-guided therapy (e.g., photodynamic therapy, electrohydraulic lithotripsy), removal of impacted pancreatic stones or stones of >5 mm, removal of intrahepatic stones, pseudocyst drainage, necrosectomy, ampullectomy, or ERCP after Whipple or Roux-en-Y bariatric surgery.

The Stanford Fluoroscopy Complexity Score was used to account for individual endoscopic interventions and their contribution to complexity [20]. To calculate this, a score ranging from 1 to 3 was assigned to each intervention based on typical intervention durations and the summation of these values denoted the final score [20]. For each procedure, one point was added for the following: ductal cannulation (for each cannulation device use), sphincterotomy, balloon dilation (for each dilation), brushing, and stone extraction (with balloon). Two points were given for the following: intraductal biopsy, guidewire and stent placement (for each stent placement), guidewire placement and choledochoscopy, electrohydraulic lithotripsy, altered surgical anatomy, mechanical lithotripsy, and stone extraction. Three points were given if mechanical lithotripsy and stone extraction were performed.

The minimum required sample size of 206 subjects was estimated based on pilot ERCP institutional data in order to detect a 1.5-minute difference in fluoroscopy time between the physician-controlled group and technologist-controlled groups. Each group had to include at least 70 patients to achieve 80% power at a two-sided significance level of 5%. Our study ultimately included 206 subjects.

Demographic and baseline patient factors were presented as mean [± standard deviation (SD)], median [interquartile range (IQR)], medians [confidence interval, (CI)], and frequencies (%). Comparisons between the groups for categorical data were done using x2 tests and Fisher's exact tests, and Wilcoxon rank-sum tests were used for non-parametric continuous data analysis. One-way analysis of variance (ANOVA) was performed to determine if there was a difference in fluoroscopy time with a particular Stanford Fluoroscopy Complexity Score (1-5, 6-10, >10). All analyses were performed using SPSS Statistics software version 22.0 (IBM, Armonk, NY), with the level of significance set at a p-value of 0.05 for all analyses. The study was approved by the organization’s Institutional Review Board.

## Results

A total of 206 patient charts were reviewed, 77 with physician-controlled (cases) and 129 with technologist-controlled (controls) fluoroscopy (Table 1). The groups did not differ in age and gender. The mean age was 62.8 (±16.6 years) for the cases and 63.8 (±17.3 years) for the controls (p=0.713). Both groups had more female than male patients with 47 (61%) and 73 (57%) women in the physician-controlled group and technologist-controlled groups, respectively (p=0.631).

**Table 1:** Experience of physicians Physicians (endoscopists) performing low-volume ERCP include those with less than 50 cases per year. Medium-volume endoscopists include those with between 50 and 200 cases per year. High-volume endoscopists include those with more than 200 cases per year. Advanced training in ERCP refers to any additional formal training in ERCP beyond a two-to-three-year general gastroenterology fellowship ERCP: endoscopic retrograde cholangiopancreatography

Physician	Number of ERCPs in study	ERCP volume	Years in practice	Advanced training in ERCP
Case 1	77	Medium	14	Yes
Control 1	65	High	16	Yes
Control 2	21	Medium	25	Yes
Control 3	6	Low	30	No
Control 4	4	Medium	6	No
Control 5	1	Low	5	No
Control 6	19	Medium	22	No
Control 7	9	Low	15	No
Control 8	4	Low	22	No

The study outcomes are presented in Table 2. The median fluoroscopy time significantly differed between the two groups with 108 seconds for physician-controlled and 146 seconds for technologist-controlled procedures (p=0.004). The difference in procedure time was also statistically significant; physician-controlled procedures were longer with a median of 2,160 seconds compared to technologist-controlled procedures, where the median procedure time was 1,950 seconds (p=0.037). Patients in the physician-controlled group had significantly more complex procedures per the Stanford Fluoroscopy Complexity Score and significantly more grade 3 procedure complexity levels than the technologist-controlled group (Table 2). In order to adjust for this difference in procedural complexity, median fluoroscopy time to procedure time ratio, median fluoroscopy time to procedure complexity level ratio, and median fluoroscopy time to Stanford Fluoroscopy Score ratios were calculated. The ratio of median fluoroscopy time to procedure time was significantly different between the two groups with 0.056 in the physician-controlled group compared to 0.082 in the technologist-controlled group (p=0.001). The ratio of median fluoroscopy time to procedure complexity level was significantly different between the two groups with 73.0 seconds in the physician-controlled cases compared to 97.0 seconds in the technologist-controlled group (p=0.002). The ratio of median fluoroscopy time to Stanford Fluoroscopy Complexity Score also significantly differed between the physician- and technologist-controlled groups with 25.5 seconds in the physician-controlled group compared to 39.3 seconds in the technologist-controlled group (p<0.001).

**Table 2:** Analysis of outcomes of physician-controlled (n=77) vs. technologist-controlled (n=129) fluoroscopy *Statistically significant difference exists between variables; **p-value not calculated, n<5 The Summation Stanford Complexity Score column for technologist-controlled is 128 because one case had a score of 0 CI: confidence interval

Variables	Physician-controlled (n=77)	Technologist-controlled (n=129)	P-value
Fluoroscopy time, seconds, median (CI)	108 (119–176)	146 (158–202)	0.004*
Procedure time, seconds, median (CI)	2,160 (2,428–3,360)	1,950 (2,041–2,595)	0.037*
Stanford Fluoroscopy Complexity Score, n (%)			
1-5	50 (64.9)	108 (83.7)	0.001*
6-10	25 (32.5)	20 (15.5)	0.005*
>10	2 (2.6)	–	**
Procedure complexity score, n (%)			
0	–	1 (0.8)	**
1	30 (39.0)	55 (42.6)	0.604
2	27 (35.1)	59 (45.7)	0.133
3	17 (22.1)	13 (10.1)	0.018*
4	3 (3.9)	1 (0.8)	**
Fluoroscopy time/procedure time, median (CI)	0.056 (0.051–0.065)	0.082 (0.084–0.103)	0.001*
Fluoroscopy time/Stanford Fluoroscopy Complexity Score, median (CI)	25.5 (24.75–37.59)	39.3 (43.49–58.74)	<0.001*
Fluoroscopy time/procedure complexity score, median (CI)	73.0 (70.74–118.16)	97.0 (105.68–140.20)	0.002*

A subgroup analysis of physicians with advanced training in ERCP was performed (Table 3). This included one physician with advanced endoscopy training who controlled fluoroscopy via a pedal (77 charts defined as cases) and two physicians with advanced endoscopy training who utilized a radiology technologist to control fluoroscopy (86 charts defined as controls). The physician-controlled group had significantly longer procedure times: 2,160 seconds vs. 1,620 seconds in the technologist-controlled group (p<0.001). The physician-controlled group had significantly fewer cases (n=50 vs. n=71 in the technologist-controlled group) in the Stanford Fluoroscopy Complexity Score of 1-5 range (p=0.026) and significantly more cases in the 6-10 range (n=25 for cases vs. n=15 for controls; p=0.012). The two groups also significantly differed in procedure complexity level of 2 (n=27 for physician-controlled vs. n=47 for technologist-controlled; p=0.012). There was a significant difference in the ratio of median fluoroscopy time to median procedure time with 0.056 for physician-controlled and 0.086 for technologist-controlled (p<0.001) as well as in the ratio of median fluoroscopy time to Stanford Fluoroscopy Complexity Score with 25.5 in the physician-controlled and 35.0 in the technologist-controlled group (p=0.001). A statistically significant difference was not found between groups with regard to the ratio of median fluoroscopy time to procedure complexity level (p=0.273).

**Table 3:** Analysis of outcomes: advanced training physician-controlled (n=77) vs. technologist-controlled (n=86) fluoroscopy *Statistically significant difference exists between variables; **p-value not calculated, n<5 CI: confidence interval

Variables	Physician-controlled (n=77)	Technologist-controlled (n=86)	P-value
Fluoroscopy time, seconds, median (CI)	108 (119–176)	131 (135–186)	0.080
Procedure time, seconds, median (CI)	2,160 (2,428–3,360)	1,620 (1,662–2,245)	<0.001*
Stanford Fluoroscopy Complexity Score, n (%)			
1-5	50 (64.9)	71 (82.6)	0.010*
6-10	25 (32.5)	15 (17.4)	0.026*
>10	2 (2.6)	–	**
Procedure complexity score, n (%)			
0	–	1 (1.2)	**
1	30 (39.0)	25 (29.1)	0.182
2	27 (35.0)	47 (54.6)	0.012*
3	17 (22.1)	12 (14.0)	0.176
4	3 (3.9)	1 (1.1)	**
Fluoroscopy time/procedure time, median (CI)	0.056 (0.051–0.065)	0.086 (0.087–0.111)	<0.001*
Fluoroscopy time/Stanford Fluoroscopy Complexity Score, median (CI)	25.5 (24.75–37.59)	35.0 (37.08–52.58)	0.001*
Fluoroscopy time/procedure complexity score, median (CI)	73.0 (70.74–118.17)	73.0 (80.05–109.13)	0.273

## Discussion

Varying estimates exist in the literature regarding the effective radiation dose associated with ERCP. The lifetime cancer risk after radiation exposure is estimated by the effective dose (ED), measured in sieverts (Sv), which is based on ionizing dose, type of radiation, and tissue weighting factors. Epidemiological research has estimated a 10% increase in cancer risk with a lifetime occupational exposure of 1 Sv [21]. A small study of 20 patients reported a mean ED of 3.1 mSv for a single diagnostic ERCP and 12.4 mSv for a single therapeutic ERCP, which is associated with a lifetime cancer risk of 1 in 6,700 and 1 in 1,700, respectively [22]. A larger, more recent study showed a median ED of 2.49 mSv [20]. Though this latter figure qualifies as low-dose radiation, the risk is stochastic and cumulative. Frequent patients may be exposed to additional radiation from other radiologic imaging or from multiple ERCPs, which is commonly indicated for therapeutic procedures. There are comparable risks of fatal cancer for physicians performing ERCP [22]. Every effort should be made to decrease cumulative radiation exposure to patients and staff [23].

In the community hospital setting, the technique of obtaining fluoroscopic imaging during ERCP varies based on a physician’s prior training and fluoroscopy control preference. There is no published data to indicate what proportion of physicians control fluoroscopy themselves using a foot pedal.

The present study revealed fluoroscopy time to be shorter when the foot pedal is controlled by the physician despite longer procedure times. This held true even after adjusting for procedure complexity; the median fluoroscopy time to procedure complexity level ratio and median fluoroscopy time to Stanford Fluoroscopy Complexity Score ratio were both significantly less in the physician-controlled fluoroscopy group. In order to further control for potential differences in technical ability when performing ERCP, we conducted a subgroup analysis to include only those physicians with advanced endoscopy training and found similar results. We hypothesize that the reason for the shorter fluoroscopy time is due to fewer communication delays when the physician is in direct control of stopping fluoroscopy. The practice at our institution is for the physician to notify the technologist when to start fluoroscopy, usually by the verbal cue, “Fluoro”. Communication delays may occur when the physician wishes to stop fluoroscopy, which could lead to unintended additional fluoroscopy time.

This study has a few limitations. Firstly, we used fluoroscopy time as the sole proxy for radiation dose; a dosimeter was not used to directly measure radiation exposure. The Society of Interventional Radiology Safety and Health Committee’s guidelines caution against using fluoroscopy time as the only metric of radiation dose due to poor correlation with other dose metrics [24]. Studies investigating radiation exposure during ERCP that have used fluoroscopy time as a metric have assumed a linear relationship to radiation exposure based on prior studies [5,13,22,25]. Dose area product (DAP) and dose at reference point (DOSERP) are more direct measures of radiation exposure that take into account other factors not reflected by fluoroscopy time alone, including patient size and position, geometry and setting of equipment, collimation, angulation, magnification, the total number of acquisition films obtained, and radiation filtration. Though fluoroscopy time is not the only determinant of radiation exposure, it has a high correlation (r: >0.9) with measured patient radiation dose and DAP with more than 90% of ERCP radiation due to fluoroscopy as opposed to X-ray pictures [16,22]. Kachaamy et al. did show a strong correlation of fluoroscopy time with DAP and DOSERP; however, there existed variability in DAP and DOSERP not accounted for by fluoroscopy time at higher radiation doses [26]. It may be prudent to include metrics such as DAP and DOSERP in future studies investigating this issue.

Our study included a relatively small number of physicians, with one case and eight controls, which may have affected the results, as variation may occur between physicians regarding the duration of fluoroscopy. Differences in individual physicians’ practice patterns may contribute to differences observed in fluoroscopy time. Future studies, with larger numbers of physicians, would be helpful to better control for this variable. As defined by previously published definitions, all endoscopists except one in this study were considered to be within the low- or medium-case volume categories. One study associated a case volume below high with increased fluoroscopy times [14]. This may be useful, as most physicians who perform ERCP are considered to be low-volume endoscopists. However, this data may not apply to physicians who perform high-volume ERCP.

## Conclusions

In summary, the results of this study suggest that physician-controlled fluoroscopy may be associated with reduced fluoroscopy time. This is a relatively simple technique to learn and adopt among physicians who are currently utilizing a technologist to obtain fluoroscopic images. This may result in reduced radiation exposure to patients and staff. Further studies are necessary to determine how widespread each of the aforementioned techniques are in clinical practice and if these results are applicable to high-volume endoscopists in an academic setting.

## References

[1] Linet MS, Slovis TL, Miller DL, Kleinerman R, Lee C, Rajaraman P, Berrington de Gonzalez A (2012). Cancer risks associated with external radiation from diagnostic imaging procedures. CA Cancer J Clin.

[2] Mettler FA Jr, Mahesh M, Bhargavan-Chatfield M (2020). Patient exposure from radiologic and nuclear medicine procedures in the United States: procedure volume and effective dose for the period 2006-2016. Radiology.

[3] Rehani MM, Ciraj-Bjelac O, Vañó E, Miller DL, Walsh S, Giordano BD, Persliden J (2010). ICRP Publication 117. Radiological protection in fluoroscopically guided procedures performed outside the imaging department. Ann ICRP.

[4] Dumonceau JM, Garcia-Fernandez FJ, Verdun FR (2012). Radiation protection in digestive endoscopy: European Society of Digestive Endoscopy (ESGE) guideline. Endoscopy.

[5] Campbell N, Sparrow K, Fortier M, Ponich T (2002). Practical radiation safety and protection for the endoscopist during ERCP. Gastrointest Endosc.

[6] Baron TH, Kozarek RA, and Carr-Locke DL (2013). ERCP, 2nd Edition. ERCP.

[7] Jowhari F, Hopman WM, Hookey L (2017). A simple ergonomic measure reduces fluoroscopy time during ERCP: a multivariate analysis. Endosc Int Open.

[8] Romagnuolo J, Cotton PB (2013). Recording ERCP fluoroscopy metrics using a multinational quality network: establishing benchmarks and examining time-related improvements. Am J Gastroenterol.

[9] Coté GA (2015). The provision of ERCP services in the United States is a radiating concern. Gastrointest Endosc.

[10] Adler DG, Lieb JG 2nd, Cohen J (2015). Quality indicators for ERCP. Gastrointest Endosc.

[11] Uradomo L, Cohen H, Fried M, Petrini J, Rehani M (2010). Radiation protection in the endoscopy suite: minimizing radiation exposure for patients and staff in endoscopy: a joint ASGE/IAEA/WGO guideline. Arab J Gastroenterol.

[12] Vehmas T (1997). Hawthorne effect: shortening of fluoroscopy times during radiation measurement studies. Br J Radiol.

[13] Kim E, McLoughlin M, Lam EC, Amar J, Byrne M, Telford J, Enns R (2010). Prospective analysis of fluoroscopy duration during ERCP: critical determinants. Gastrointest Endosc.

[14] Jorgensen JE, Rubenstein JH, Goodsitt MM, Elta GH (2010). Radiation doses to ERCP patients are significantly lower with experienced endoscopists. Gastrointest Endosc.

[15] Heyd RL, Kopecky KK, Sherman S, Lehman GA, Stockberger SM (1996). Radiation exposure to patients and personnel during interventional ERCP at a teaching institution. Gastrointest Endosc.

[16] Uradomo LT, Lustberg ME, Darwin PE (2006). Effect of physician training on fluoroscopy time during ERCP. Dig Dis Sci.

[17] Uradomo LT, Goldberg EM, Darwin PE (2007). Time-limited fluoroscopy to reduce radiation exposure during ERCP: a prospective randomized trial. Gastrointest Endosc.

[18] Buls N, Pages J, Mana F, Osteaux M (2002). Patient and staff exposure during endoscopic retrograde cholangiopancreatography. Br J Radiol.

[19] Tsalafoutas IA, Paraskeva KD, Yakoumakis EN, Vassilaki AE, Maniatis PN, Karagiannis JA, Koulentianos ED (2003). Radiation doses to patients from endoscopic retrograde cholangiopancreatography examinations and image quality considerations. Radiat Prot Dosimetry.

[20] Liao C, Thosani N, Kothari S, Friedland S, Chen A, Banerjee S (2015). Radiation exposure to patients during ERCP is significantly higher with low-volume endoscopists. Gastrointest Endosc.

[21] Hendee WR (1992). Estimation of radiation risks. BEIR V and its significance for medicine. JAMA.

[22] Larkin CJ, Workman A, Wright RE, Tham TC (2001). Radiation doses to patients during ERCP. Gastrointest Endosc.

[23] Naidu LS, Singhal S, Preece DE, Vohrah A, Loft DE (2005). Radiation exposure to personnel performing endoscopic retrograde cholangiopancreatography. Postgrad Med J.

[24] Stecker MS, Balter S, Towbin RB (2009). Guidelines for patient radiation dose management. J Vasc Interv Radiol.

[25] Boix J, Lorenzo-Zúñiga V (2011). Radiation dose to patients during endoscopic retrograde cholangiopancreatography. World J Gastrointest Endosc.

[26] Kachaamy T, Harrison E, Pannala R, Pavlicek W, Crowell MD, Faigel DO (2015). Measures of patient radiation exposure during endoscopic retrograde cholangiography: beyond fluoroscopy time. World J Gastroenterol.

